# Identification of Protein Quality Markers in Toad Venom from *Bufo gargarizans*

**DOI:** 10.3390/molecules28083628

**Published:** 2023-04-21

**Authors:** Meiyun Yang, Weiwei Huan, Guobing Zhang, Jie Li, Fengyan Xia, Rabia Durrani, Wei Zhao, Jidong Lu, Xinmeng Peng, Fei Gao

**Affiliations:** 1 Zhejiang Provincial Key Laboratory of Resources Protection and Innovation of Traditional Chinese Medicine, Zhejiang A&F University, Hangzhou 311300, China; 2 Zhejiang Provincial Key Laboratory of Chemical Utilization of Forestry Biomass, College of Chemistry and Materials Engineering, Zhejiang A&F University, Hangzhou 311300, China; 3 Department of Pharmacy, Zhejiang Province People’s Hospital, Hangzhou 310014, China; 4 The Second Affiliated Hospital, Zhejiang University School of Medicine, Hangzhou 313000, China; 5 State Key Laboratory of Subtropical Silviculture, Zhejiang A&F University, Hangzhou 311300, China

**Keywords:** *Bufo gargarizans*, ear-side glands, toad venom, proteomics, extracellular proteins, antimicrobial activity, analgesic activity, quality markers

## Abstract

Toad venom is a traditional Chinese medicine with high medicinal value. The existing quality evaluation standards of toad venom have obvious limitations because of the lack of research on proteins. Thus, it is necessary to screen suitable quality markers and establish appropriate quality evaluation methods for toad venom proteins to guarantee their safety and efficacy in clinical applications. SDS-PAGE, HPLC, and cytotoxicity assays were used to analyze differences in protein components of toad venom from different areas. Functional proteins were screened as potential quality markers by proteomic and bioinformatic analyses. The protein components and small molecular components of toad venom were not correlated in content. Additionally, the protein component had strong cytotoxicity. Proteomics analysis showed that 13 antimicrobial proteins, four anti-inflammatory and analgesic proteins, and 20 antitumor proteins were differentially expressed extracellular proteins. A candidate list of functional proteins was coded as potential quality markers. Moreover, Lysozyme C-1, which has antimicrobial activity, and Neuropeptide B (NPB), which has anti-inflammatory and analgesic activity, were identified as potential quality markers for toad venom proteins. Quality markers can be used as the basis of quality studies of toad venom proteins and help to construct and improve safe, scientific, and comprehensive quality evaluation methods.

## 1. Introduction

The amphibian family, Bufonidae, is one of the oldest living groups on Earth. It is characterized by the presence of parotid macro glands behind the eyes and ears, which are primarily known for their toxic secretions [1,2,3,4]. The toxins are used as a chemical defense against predators and microbial infection [5,6]. Toad venom, as a traditional Chinese medicine with high medicinal value, is the dried product of toxic secretions of the ear-side glands and skin glands of *Bufo bufo gargarizans* Cantor or *Bufo melanostictus* Schneider. Toad venom has been widely applied to treat diseases for thousands of years in Asia, especially in China, Korea, and Japan [7,8].

Toad venom possesses great potential as a pharmaceutical therapy because of its rich active ingredients. More than 100 such chemical components have been identified in the secretions from different species of toads, including bufadienolides, indole alkaloids, biogenic amines, steroids, peptides, and proteins [9,10]. According to modern medical analysis, toad venom has a variety of pharmacological activities such as cardiotonic, anesthesia, detoxification, analgesia, antimicrobial, antitumor, and immune enhancement activities [11,12,13]. There are 88 varieties of Chinese patent medicines containing toad venom in the “Ministry Standards” and “Pharmacopoeia of the People’s Republic of China”, including Shexiang Baoxin pills, Niuhuang Xiaoyan pills, and Liushen pills.

Chemical research on toad venom domestically and abroad is relatively in-depth, and early research focused on two types of small molecular components: bufadienolides and indole alkaloids, such as bufalin, cinobufagin, and bufothionine [8,12]. Jo et al. found that the potential anticancer mechanism of cinobufalin ligand in oral squamous cell carcinoma is the downregulation of chloride channel protein 1(ANO1) [14]. Zheng et al. clarified the anti-inflammatory effects of bufadienolides from toad venom and indicated that gammabufotalin is expected to be a novel therapeutic agent for inflammatory diseases [15]. Two new polyamine alkaloids, together with four known alkaloids, were isolated from the *Bufo viridis* toad venom [16]. Nowadays, there are still a large number of studies on the small molecular components of toad venom but fewer on other components.

In recent years, biological macromolecular drugs, such as proteins and peptides, have attracted increasing attention because of their high activity, strong specificity, low toxicity, and clear biological functions [9]. To date, approximately 2000 peptides with a high diversity of biological activities belonging to more than 100 peptide families have been identified in skin secretions from amphibians, such as antimicrobial peptides, neuropeptides, myotropic peptides, and angiotensins [10,17,18,19,20]. For example, Chang et al. showed that a peptide homolog of prokineticin from the venom of the frog *Amolops Jingdongenesis* is a potent wound-healing regulator [21]. In addition, many non-amphibians possess potent bioactive peptides. The anticoagulant component phospholipase A2 was found in *Deinagkistrodon acutus* venom [22]. Conotoxin is a peptide drug that can potentially be used as an antiepileptic [23]. A novel toxin, LCTX-F2, with coagulation-promoting activity, was identified in the venom of the spider *Lycosa singoriensis* [24]. Hirudin, a thrombin inhibitor, is considered a promising therapeutic agent for renal interstitial fibrosis [25]. Moreover, melittin is widely known for its various pharmacological actions [26].

In the past years, researchers have gradually focused on the macromolecular components of toad venom. Proteins with antioxidant, catalytic, and transport activities and polypeptide components related to antitumor, antimicrobial, and analgesic properties have been identified from toad venom [2,27]. For instance, two Bombinin peptides isolated from skin secretions of the Oriental Fire-bellied toad *Bombina orientalis* exhibit antimicrobial and anticancer activities [28]. Gao et al. identified the antimicrobial peptide cathelicidin-Bg, antitumor components, and angiogenesis-inhibiting peptides from the venom of the toad *B. gargarizans* [2,20,27]. There were 23 unique peptides that showed defensive properties in the deionized water extract of the parotoid gland secretion from *B. gargarizans* [11]. However, research on proteins and peptides from the venom of the toad *B. gargarizans* is insufficient.

Toad venom is listed as one of the 28 types of poisonous traditional Chinese medicines, and a more comprehensive safety evaluation is needed. Studies have confirmed significant differences in active compositions and comprehensive qualities of toad venom (*B. gargarizans*) under different geographical conditions [29,30]. With the rising price of toad venom, increasing falsification and adulteration technology has resulted. Furthermore, more and more proteins and peptides with great medicinal potential have been discovered in toad venom [11], and the limitations of the existing quality evaluation methods and standards of toad venom are highlighted. “Pharmacopoeia of the People’s Republic of China” (2020 edition) stipulates the quality standards for toad venom, the total amount of bufalin, cinobufagin, and resibufogenin in dried bufalin samples should not be less than 7.0%. The current legal quality evaluation only includes small molecular components, and there is a lack of research on the quality of the protein components. Therefore, quality markers can be screened from protein components and incorporated into the quality control system [31,32], and a scientific and comprehensive quality evaluation method can be established to ensure its safety and efficacy in clinical applications.

Currently, underlying component content and profiling, many useful methods have been developed to identify proteins and peptides at the transcriptomic, peptidomic, or genomic level to analyze the active substances, reveal the quality markers in medicinal materials, and perform an overall quality evaluation through bioinformatics and validation [33,34,35,36]. Here, transcriptomics and tandem mass tag (TMT) proteomics were used to analyze differences and identify quality markers in protein components of toad venom from different regions. We explored combinations of techniques to identify potential protein quality markers and reveal the protein composition of *B. gargarizans*, which could facilitate the discovery of novel toad proteins and peptides and provide a basis for the exploration of safety, scientific, and comprehensive quality standards of toad venom.

## 2. Results

### 2.1. Morphology of the Ear-Side Gland in the Toad

**Figure 1:**
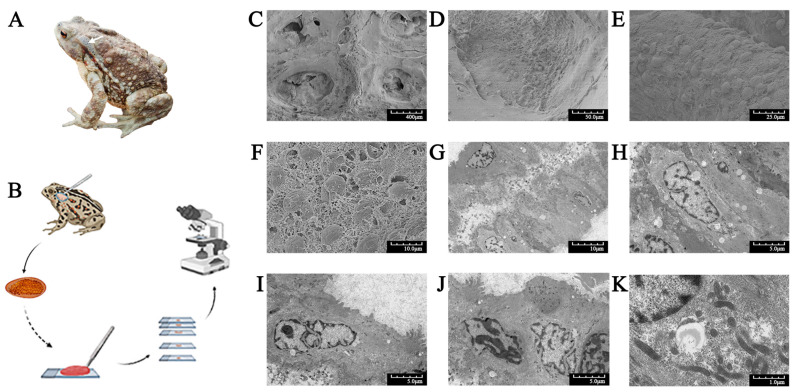
Morphology of *Bufo gargarizans* and electron microscopy of its ear-side glands. (**A**) Dorsal view of an adult specimen. The white arrow indicates the ear-side gland. (**B**) The process of collecting glands, making slides, and microscopic observation. (**C**) The honeycomb-like structure of a gland. (**D**) A poison gland containing large poison granules. (**E**) Poison granules immersed in the syncytial cytoplasm matrix. (**F**) Poison granules with shrunken surfaces and syncytial cytoplasm matrix with a spongy appearance. (**G**) A monolayer of acini cells around a characteristic central lumen. (**H**–**J**) A monolayer of secretory cells. There was no clear boundary between the cells, and the nuclei were obvious, with clear edges and folds. (**K**) The syncytial cytoplasm matrix containing high numbers of organelles. Scanning electron microscopy (**C**–**F**). Transmission electron microscopy (**G**–**K**).

The ear-side gland is a round and plump glandular structure (Figure 1A,B), which is the most ubiquitous macro gland in toads of the Bufonidae family [3]. This gland plays a key role in chemical defense against predators and microorganisms [4].

Scanning electron microscopy observation revealed that the internal transverse section of the gland presented an internal honeycomb-like cluster (Figure 1C), which clearly showed large poison granules lodged within the cytoplasm matrix (Figure 1D–F). Interwoven reticular structures were observed between granules, and the syncytial cytoplasm matrix had a spongy appearance (Figure 1F).

Transmission electron microscopy investigations showed acini consisting of a monolayer of secretory cells organized around a characteristic central lumen (Figure 1G), and no clear boundary was present between the cells (Figure 1G–J). The membrane was pleated and formed a microvillus brush border. The syncytial nuclei were obvious, with clear edges and folds (Figure 1H–J). Moreover, the organelles and secretion granules involved in poison synthesis and storage were dispersed and surrounded the nucleus throughout the syncytial cytoplasm (Figure 1K).

### 2.2. Composition of Venom from Toads from Different Areas

**Figure 2:**
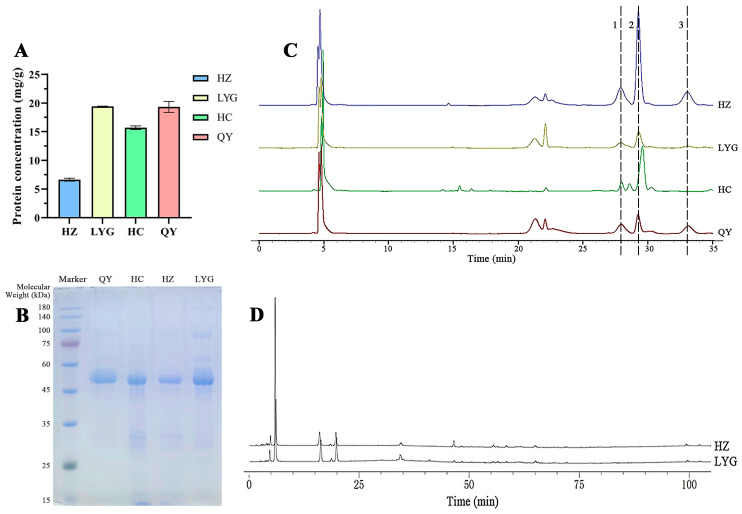
Composition differences between small molecular components and protein components of the venom of toads from different areas. (**A**) Protein quantification of toad venom from four areas. (**B**) Sodium dodecyl sulfate-polyacrylamide gel electrophoresis results of toad venom from four areas. (**C**) HPLC fingerprints of small molecular components of toad venom from four areas. 1: bufalin, 2: cinobufagin, 3: resibufogenin. (**D**) HPLC fingerprints of protein components of venom of toads from Hanzhong (HZ) and Lianyungang (LYG).

The HPLC results showed differences in the quality of venom of toads from different areas (Figure 2). According to the method recommended by the “Pharmacopoeia of the People’s Republic of China”, the areas of the characteristic peaks (bufalin, cinobufagin, and resibufogenin) of the four samples were found to be significantly different (Figure 2C). Among them, the sum of characteristic peak areas of the Hanzhong (HZ) specimens was the largest, up to 3,070,161 mV, and the content of small molecular components was the highest; the sum of the peak areas of the Lianyungang (LYG) specimens was the smallest, only 485,306 mV, with a low small molecular component content.

The protein component is another active substance of toad venom. The total protein content of toad venom varied in different areas (Figure 2A). Among them, samples from toads from HZ and LYG showed the largest difference in total protein content. The LYG specimens had the highest total protein content, while the total protein content of the HZ specimens was the lowest (19.42 mg/g and 6.63 mg/g, respectively).

The SDS-PAGE results revealed that the protein profile of toad venom similarly varied in toads from different areas (Figure 2B). An obvious main protein band existed between 50 and 55 kDa, whereas another less intense band occurred in the range of 80–100 kDa. In addition, the bands in the LYG and Qinyuan (QY) specimens were darker, and the LYG specimens also had obvious bands near 30 kDa and 60 kDa.

As shown in Figure 2D, HPLC analysis of the protein extract showed more than 10 different peaks. Under the same injection concentration (1 mg/mL) and retention time, the HZ and LYG specimens showed corresponding chromatographic peaks; however, the peak area of the LYG specimens was larger, and the protein content was higher. The protein content of toad venom showed trends opposite to those of bufalin, cinobufagin, and resibufogenin. The small molecular component and protein component of HZ and LYG were the most different.

### 2.3. Cell Inhibitory Activity of Venom Proteins of Toads from Different Areas

**Figure 3:**
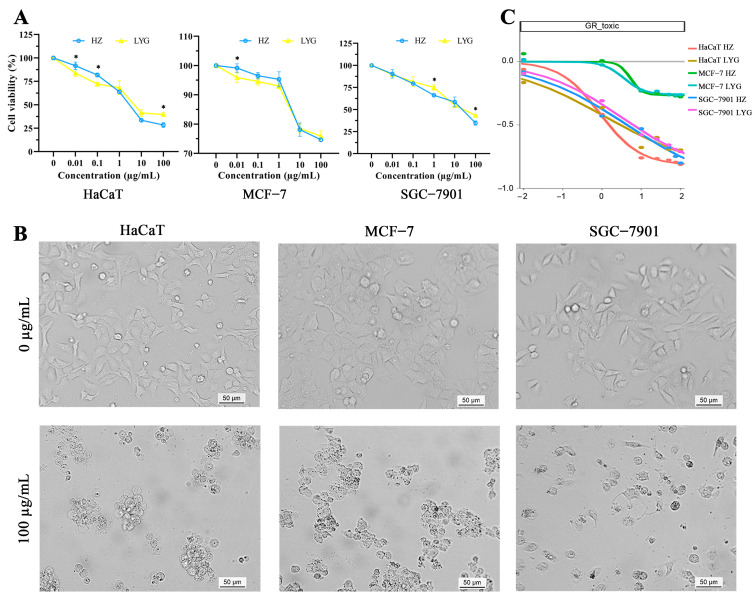
Morphological changes and dose-dependent effects of toad venom specimens from HZ and LYG on the viability and growth rate inhibition (GR) of HaCaT, MCF–7, and SGC–7901 cells. (**A**) Cytotoxicity of the HZ and LYG specimens in three types of cells was determined by an MTT assay. Values are presented as means ± SD, N = 5. * *p* < 0.05 compared between the HZ and LYG specimens at the same concentration. (**B**) Morphological changes in the three types of cells following treatment with protein components of toad venom. (**C**) GR of the HZ and LYG specimens in three types of cells.

To more accurately analyze the difference in protein cytotoxicity in venom extracts between toads from HZ and LYG and examine the effect of those proteins on the growth of human cells (HaCaT, MCF-7, and SGC-7901 cells), an MTT assay and growth rate inhibition (GR) were performed (Figure 3). The results of the MTT assay and GR indicated that both HZ and LYG toad venom protein extracts had strong cytotoxicity (Figure 3A), especially for HaCaT and SGC-7901 cells. The two extracts were found to reduce HaCaT, MCF-7, and SGC-7901 cell viability in a dose-dependent manner in the concentration range of 0.01–100 µg/mL. Given a range of drug concentrations, growth rate inhibition of toad venom proteins between 0 and −1 indicates a dose-dependent cell death (Figure 3C). At the same protein concentration, the two toad venom proteins had similar cytotoxicity, so their protein composition could be similar. High concentrations of extracts induced high proteotoxicity and caused the three types of cells to exhibit decreased numbers and abnormal morphology and easily grow in clumps (Figure 3B).

Toad venom protein toxicity and activity have not been shown to be related to small molecular components such as bufalin, cinobufagin, and resibufogenin. Because of the lack of studies on the toxicity and activity of proteins, the existing quality standards cannot be used to adequately evaluate the quality of toad venom.

### 2.4. Proteomic Characterization of Toad Venom

**Figure 4:**
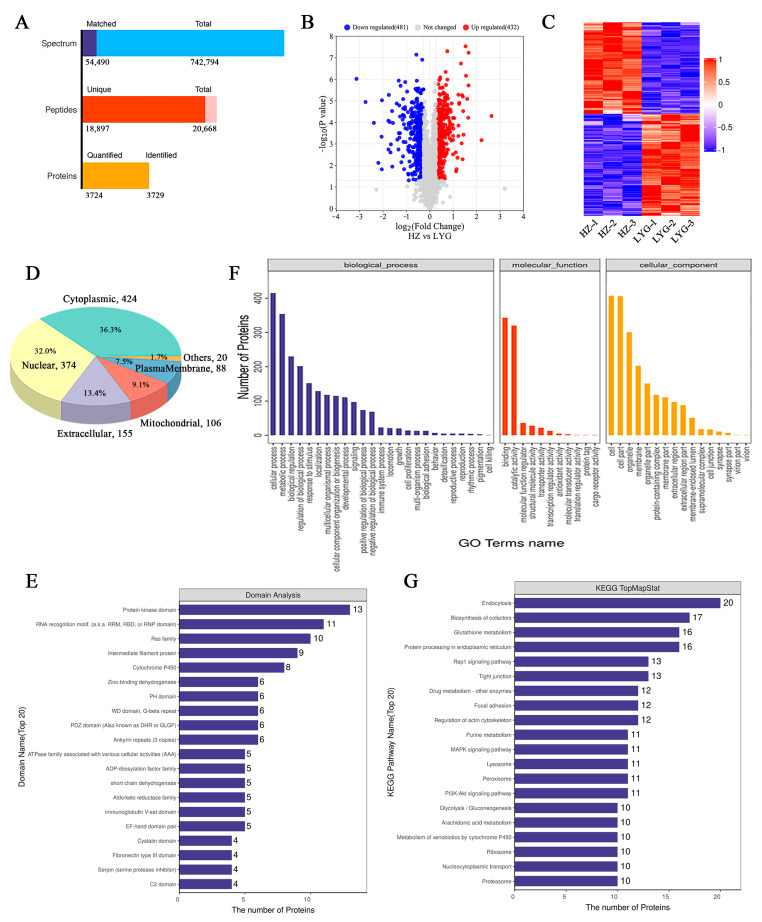
Proteome differences between the HZ and LYG samples. (**A**) Protein identification and quantitative results. (**B**) Volcano plot analysis of all differentially expressed proteins (DEPs). (**C**) Heatmap of all proteins. (**D**) Subcellular pie chart of all DEPs. (**E**) Domain analysis of all DEPs (Top20). (**F**) Statistical distribution of all DEPs under GO annotation. (**G**) KEGG pathway annotation statistical map of all DEPs (Top20).

Transcriptomic and proteomic methods were used to further investigate the protein composition of toad venom, and all proteins and differentially expressed proteins (DEPs) in HZ and LYG specimens were identified. Using the transcriptome database for protein identification, a total of 742,794 spectra, 18,897 unique peptides, and 3724 quantified proteins (unique peptides ≥ 1) were identified based on TMT quantitative proteomics (Figure 4A). Among the quantified proteins, the total number of DEPs (fold change ≥ 6/5 or ≤5/6) was 913 (Figure 4B). All DEPs of each group were analyzed in the form of a clustering heat map; the good repeatability of samples indicated that the data were highly reliable (Figure 4C).

All DEPs were analyzed to determine the subcellular localization using CELLO (http://cello.life.nctu.edu.tw/, accessed on 13 July 2022); 36.3% of DEPs were cytoplasmic, 32.0% were nuclear, and 13.3% were extracellular (Figure 4D). The domain prediction software InterProScan (version 5.52-86.0-64; EMBL-EBI, Cambridgeshire, UK) was used to analyze the annotation of functional domains of DEPs. The greatest enrichment of DEPs was found in the protein kinase domain (Figure 4E). The most enriched categories of DEPs were cellular process, metabolic process, regulation of biological process and other important biological processes, catalytic activity, structural molecule activity, transporter activity, and other molecular functions; the localization proteins included organelle part, membrane part, and extracellular region part (Figure 4F). All DEPs were annotated according to the KEGG pathway, and the number of DEPs was counted. Among them, the top pathways with the largest number of differential proteins were endocytosis, biosynthesis of cofactors, glutathione metabolism, and protein processing in the endoplasmic reticulum (Figure 4G).

### 2.5. Identification of Toad Venom Protein Quality Marker

**Figure 5:**
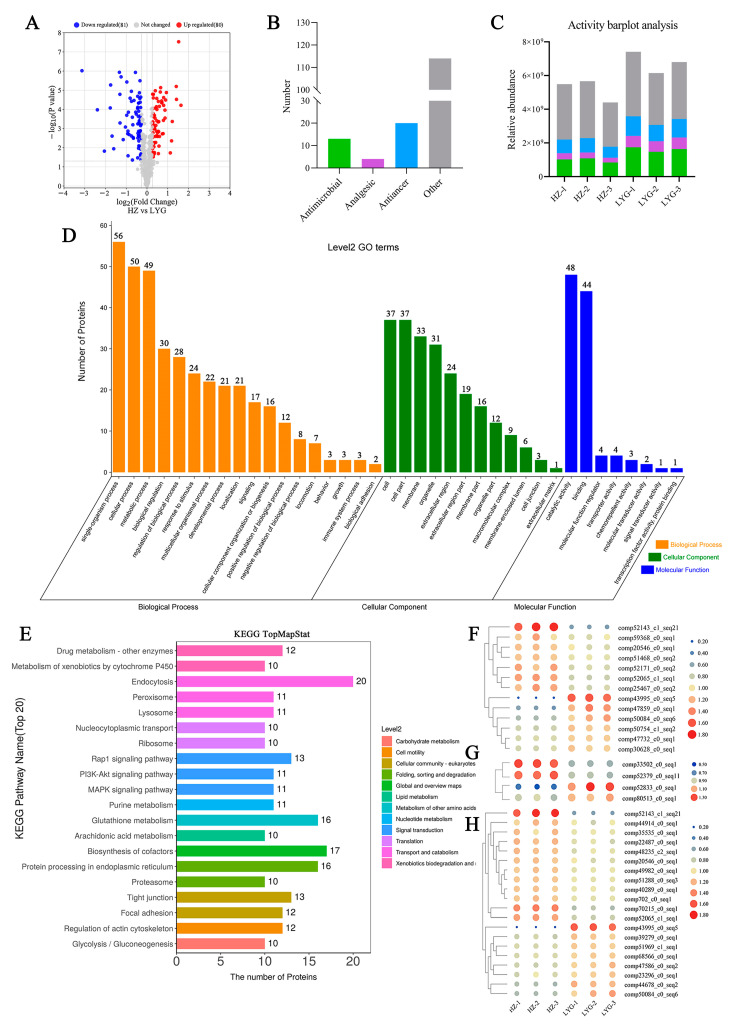
Functional protein analyses and screening for quality markers of extracellular differentially expressed proteins (eDEPs). (**A**) Volcano plot analysis. (**B**) Numbers of the three types of functional proteins among eDEPs. (**C**) Relative abundance analysis of the three types of functional proteins among eDEPs. (**D**) Statistical distribution of eDEPs under GO annotation. (**E**) KEGG pathway annotation statistical map of eDEPs (Top20). (**F**–**H**) Differential expression heat maps of antimicrobial, anti-inflammatory and analgesic, and antitumor active proteins, respectively.

Toad venom is a well-known secretory substance with antitumor, antimicrobial, analgesic, anti-inflammatory, and other pharmacological activities [10,11,12,13]. We focused on the extracellular functional proteins that were differentially expressed in the HZ and LYG specimens. The total number of extracellular proteins was 436 (Figure 5A), including 161 extracellular differentially expressed proteins (eDEPs), 80 upregulated and 81 downregulated). Among all the eDEPs, there were 13, 4, and 20 proteins with antimicrobial, anti-inflammatory and analgesic, and antitumor activity, respectively (Figure 5B). Compared with the HZ specimens, three types of functional proteins showed higher relative abundance in the LYG specimens (Figure 5C). These proteins were enriched mainly in the following categories: single-organism process, cellular process and metabolic process, membrane, organelle, and extracellular region part, and binding and catalytic activity (Figure 5D). According to the KEGG analysis (Figure 5E), endocytosis and biosynthesis of cofactors were enriched among the eDEPs.

To identify proteins related to three types of biological activity (Figure 5F–H), 13 (7 upregulated and 6 downregulated) antimicrobial DEPs, 4 (2 upregulated and 2 downregulated) anti-inflammatory and analgesic DEPs, and 20 (12 upregulated and 8 downregulated) antitumor activity DEPs were screened based on a comparison of the HZ and LYG specimens. The protein identification information is shown in Table 1, and more information about these proteins is shown in Table S1.

Among the high-component functional substances of eDEPs, the most abundant antimicrobial and antitumor proteins were Comp43995_c0_seq5 (E3 ubiquitin-protein ligase makorin-2, Makorin-2) and Comp52143_c1_seq21 (Lysozyme C-1). Comp52833_c0_seq1 (secretory phospholipase A2 DsM-S1, DsM-S1) and Comp33502_c0_seq1 (Neuropeptide B isoform 2, NPB) were the most abundant anti-inflammatory and analgesic proteins. Additionally, these proteins showed the most significant differential expression between the HZ and LYG specimens. Therefore, these proteins can be further studied as potential quality markers of toad venom protein.

### 2.6. Functional Validation of Toad Venom Protein

Table 1 showed that Lysozyme C-1 had antimicrobial activity and NPB had anti-inflammatory and analgesic activity. Lysozyme C-1 and NPB were obtained for functional validation experiments by fusion expression in vitro and direct synthesis, respectively.

**Figure 6:**
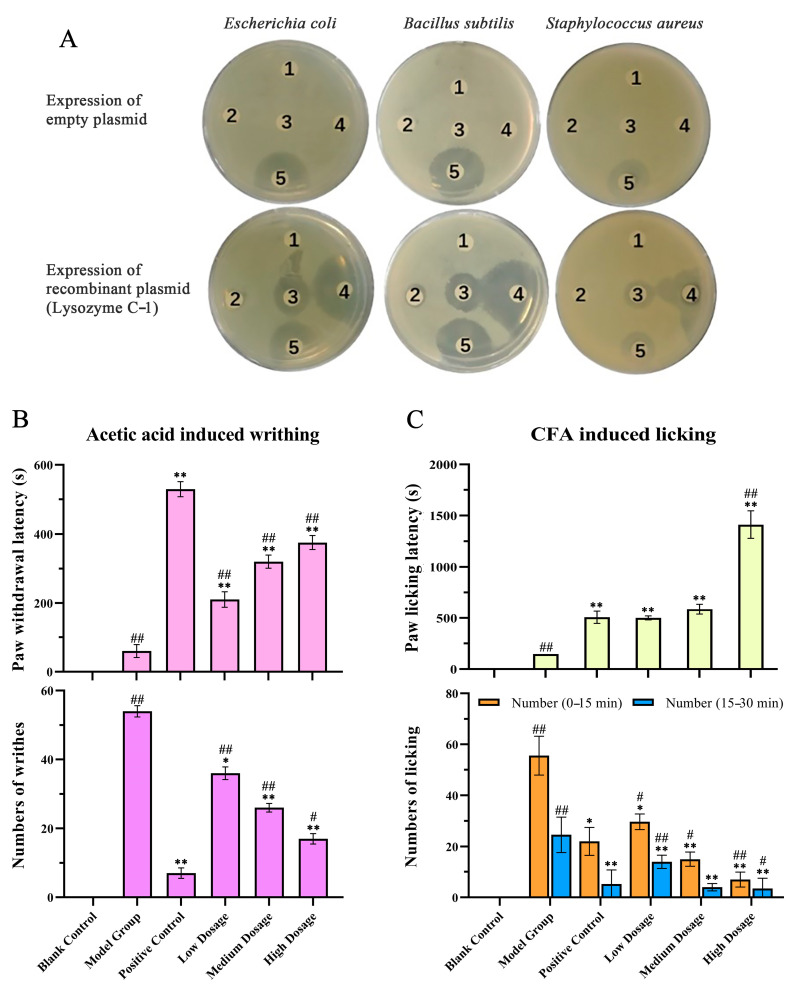
Activity validation of antimicrobial, anti-inflammatory, and analgesic components. (**A**) antimicrobial activity of a fusion protein solution containing lysozyme. (Note: 1: phosphate-buffered saline (PBS) group; 2: protein concentration 0.01 μg/μL; 3: protein concentration 0.1 μg/μL; 4: protein concentration 1 μg/μL; 5: antibiotic group 0.5μg/ μL.). (**B**,**C**) Anti-inflammatory and analgesic activity of the NPB polypeptide. (**B**) Acetic acid-induced writhing latency and duration over 15 min in mice. (**C**) Complete Freund’s adjuvant (CFA)-induced paw licking latency and amount of licking within the first phase (0–15 min) and the second phase (15–30 min) in rats. Note: The results indicate the mean ± SD of each group of experiments, N = 5; * *p* < 0.05, ** *p* < 0.01 compared with the model group, # *p* < 0.05, ## *p* < 0.01 compared with the positive control group.

An antimicrobial activity assay (Figure 6A) showed that larger dose-dependent inhibition zones appeared on LB plates of *Escherichia coli*, *Bacillus subtilis*, and *Staphylococcus aureus* after treatment with protein solutions containing lysozyme than those in the control groups. Compared with the positive antibiotic control group, we found that the antimicrobial zones around the filter papers with 1 µg/µL of fusion protein solution were larger, indicating that the bactericidal effect of high-concentration (1 µg/µL) Lysozyme C-1 solution was similar to that of 0.5 μg/µL ampicillin-kanamycin.

As shown in Figure 6B,C, in the anti-inflammatory and analgesic activity assays, the blank group did not show writhing or licking movements; therefore, there was no latency time. The latency times of the positive control group and NPB groups were longer than that of the model group, and the writhing (Figure 6B) and licking (Figure 6C) latency times were dependent on the concentrations of the agents. The model group showed the most writhing and licking, and aspirin and NPB significantly reduced the acetic acid-induced writhing times in mice and the Complete Freund’s adjuvant (CFA)-induced licking times in rats (*p* < 0.01).

## 3. Discussion

An analysis of toad gland poison reported that 25% to 35% of the dry weight of toad venom consists of proteins [4]. Although they are present in considerable amounts in toad venom, proteins have received little attention compared with other compounds; therefore, we have examined proteins in toad venom in-depth.

Ultrastructure studies of the toad ear-side glands that secrete venom showed that many giant poison glands formed characteristic honeycomb-like structures, which was consistent with previous reports. The syncytium had a large nucleus, and abundant organelles were involved in protein synthesis and storage in these glands [1,4,11,13], including the endoplasmic reticulum, Golgi bodies, ribosomes, and secretory granules. Large amounts of secretions were stored in granules immersed in the cytoplasmic matrix, which were used as a chemical defense against predators and microorganisms [3,4].

The major SDS-PAGE bands and characteristic HPLC peaks showed that protein fractions of higher intensities were consistent in toad venom, likely because of the structural stability of some peptides in toad venom [37]. The differences in the color depth of the bands and the size of the chromatographic peak areas indicated that the HZ and LYG specimens showed the greatest differences in protein quality. The total protein content of the LYG specimens was highest, and that of the HZ specimens was lowest, consistent with the protein content determination. Small molecular components of toad venom were analyzed according to the existing quality standards, showing the HZ specimens had the highest active constituent content and that the LYG had the lowest active constituent content, which was contrary to the toad venom protein study results.

The cytotoxicity and GR of the proteins extracted from the HZ and LYG specimens on human cells were analyzed. In the concentration range of 0.01–100 µg/mL, both protein extracts showed a dose-dependent inhibitory effect on cell growth. The two extracts had similar inhibitory effects on cells at the same protein concentration, so their protein composition could be similar. However, the LYG specimens had higher protein levels than the HZ specimens for the same mass of toad venom. Using the same amount of sample, the cell viability of the LYG samples was lower, the toxicity was stronger, and the effect of the active ingredient was greater than that of the HZ samples. Furthermore, the GR value relates directly to the response phenotype, and the inhibitory effect of venom on cell growth was also manifested by morphological changes. At high concentrations, three types of cells decreased in number and were abnormal in morphology. These results indicated that toad venom proteins exhibit toxicity and biological activity, which is worthy of further study.

The above results showed trends opposite of the content between the small molecular components and the protein components in toad venom. This finding indicated that the quality standard of the small molecular components of toad venom could not simply be used to assess proteins. Safety standards and scientific and comprehensive quality evaluation methods are lacking, and there are obvious limitations of the existing quality evaluation standards.

However, the enormous complexity and considerable amount of proteins present great challenges to the study of toad secretions [11], and the identity and actual function of toad venom proteins are still quite controversial [4]. In combination with proteomics and transcriptomics [38,39], the composition of active proteins in toad venom was clarified to further investigate their potential as quality markers. We focused on eDEPs because toad venom is a secretory substance. A total of 161 eDEPs were uniquely altered: 80 were upregulated, and 81 were downregulated. These preliminary results indicated that secreted protein is a rich source for studying quality markers of toad venom.

The proteomics results were compared using self-built databases of antimicrobial, anti-inflammatory, and analgesic proteins, and 13 (7 upregulated and 6 downregulated) antimicrobial eDEPs, 4 (2 upregulated and 2 downregulated) anti-inflammatory and analgesic eDEPs, and 20 (12 upregulated and 8 downregulated) antitumor activity eDEPs were identified. Then, Comp43995_c0_seq5 (Makorin-2), Comp52143_c1_seq21 (Lysozyme C-1), Comp52833_c0_seq1 (DsM-S1), and Comp33502_c0_seq1 (NPB) were screened as potential quality markers of toad venom protein.

Lysozymes and lysozyme-like enzymes with antimicrobial effects can be used as antimicrobial agents [40,41]. Lysozyme C-1 has been reported to play an antimicrobial role in immune responses and microbiota digestion in mosquitoes [42]. Additionally, many experimental studies have confirmed the anticancer activity of lysozymes in a variety of tumors [43]. In the central nervous system, members of the NPB/W signaling system play a role in modulation of inflammatory pain and neuroendocrine functions [44,45,46]. Morphine, NPB23, and NPW23 can synergistically relieve inflammatory and neuropathic pain by activating different receptors [46]. However, the E3 ubiquitin-protein ligase Makorin-2 has rarely been examined in antimicrobial and antitumor studies, and secretory phospholipase A2 DsM-S1 has rarely been investigated in anti-inflammatory and analgesic studies. Therefore, the functional activities of Lysozyme C-1 and NPB can be further validated to determine whether they can be used as quality markers.

The antimicrobial assay of Lysozyme C-1 illustrated that it had an inhibitory effect on three microorganisms, and the effect was cell type-dependent as well as dose-dependent. It was speculated that lysozyme displays antimicrobial activity in Gram-negative bacteria and Gram-positive bacteria. The animal experiment results revealed that NPB had an evident analgesic effect compared with the model group and positive control group and produced a dose-dependent anti-inflammatory and analgesic response. These functional proteins indeed have corresponding functions. In addition, antitumor protein fractions from toad venom have previously been studied; a bFGF-immobilized affinity column was used to capture three potential peptides that could inhibit the proliferation of bFGF-dependent cells [2]. Finally, Lysozyme C-1 and NPB can be used as quality markers of toad venom protein in the future.

In summary, many proteins and peptides in toad venom have significant physiological activity and pharmaceutical potential [9,10,11,12,13]. Lysozyme C-1 and NPB are highly active protein components and were determined to be quality markers that can enrich the quality evaluation criteria of toad venom and improve the quality control system. Currently, the emergence and spread of antimicrobial resistance [47] and analgesics also have strong toxicity and side effects [48], which seriously affects people’s health and life. Antimicrobial peptide Lysozyme C-1 and analgesic peptide NPB can be used as potential high-quality drugs. We can study the protein structure and clarify the activity mechanism of Lysozyme C-1 and NPB, and we can also biomodify them to make them more active and functional.

However, there were some limitations to our study. First, the numbers and sources of samples were small, which should be increased to reduce the effects of experimental error and increase the reliability of the results. Second, not all DEPs were secretory proteins; most DEPs were constitutive proteins. It should be determined whether this constitutive protein excess occurred because of the excessive collection of certain body tissues during toad venom collection or the specific secretion mode of toad venom. In addition, the correlation between the high extracellular content protein and functional proteins can be analyzed, and more easily detected substances can be selected as markers to analyze the quality of toad venom proteins. Therefore, further research is needed to overcome the limitations of this study.

## 4. Materials and Methods

### 4.1. Toads and Toad Venom Samples

Ten toad specimens were collected from Zhejiang A&F University, Lin’an, Hangzhou, Zhejiang Province, in September 2021.

Fresh venom of toads from different areas, such as Hanzhong (HZ), Lianyungang (LYG), Hanchuan (HC), and Qinyuan (QY), were collected from June to August 2020. Toad venom is collected with special metal pliers (mostly made of copper or aluminum). The secretion of the ear gland was collected first, and then the secretion of the skin gland was collected and stored at −80 °C. Toad venom from each area was a mixture of secretions from 40 toads. All experimental toads were identified as *B. gargarizans* by experts engaged in animal taxonomy.

### 4.2. Ultrastructure of the Glands

Scanning electron microscopy. The ear-side glands of the sacrificed toads were wiped with 75% ethanol solution and cut after squeezing secretions. The glands were rinsed with PBS (0.01 M, pH 7.4) and then fixed in 2.5% glutaraldehyde fixative mixed with 1% osmium tetroxide. After dehydration in a gradient of ethanol solutions, the samples were dried in a critical point apparatus (Hitachi Model HCP-2, Tokyo, Japan) and then coated and examined under a scanning electron microscope (Hitachi Model SU-8010, Tokyo, Japan) [4].

Transmission electron microscopy. The glands were fixed and dehydrated following the same protocol described above. After dehydration, the samples were embedded in Spurr embedding medium and acetone and then sectioned with an ultramicrotome (Leica EM UC7, Frankfurt, Germany). Ultrathin sections were immersed in lead citrate and uranyl in 50% ethanol and examined under a transmission electron microscope (Hitachi H-7650, Tokyo, Japan).

### 4.3. Total Protein Extraction and Content Determination

The total protein of toad venom was extracted using an acetone precipitation method [20]. Fresh toad venom was extracted with PBS (0.01 M, pH 7.4) for approximately 4 h. The samples were centrifuged (12,000× *g*, 15 min, 4 °C), and the supernatant was collected; this process was repeated three times. After adding pre-chilled acetone, the protein precipitate was collected, re-dissolved in PBS, and stored at −80 °C for further analysis.

The protein concentration was measured using a Pierce BCA Protein Assay Kit (Thermo Fisher Scientific, Waltham, MA, USA) according to the manufacturer’s instructions [49,50]. The protein content was determined using the standard formula optical density = 1.2316x + 0.0693 (*R*^2^ = 0.9988).

### 4.4. SDS-PAGE Analysis

Protein from each sample (30 µg) was mixed with 5× loading buffer and boiled for 5 min. The proteins were separated on a 10% SDS-PAGE gel (constant voltage 180 V, 60 min). Protein bands were visualized by Coomassie Blue R-250 staining [51,52].

### 4.5. HPLC Analysis

A Waters system (Waters e2695 Separations Module and Waters 2998 PDA Detector, Milford, MA, USA) with an A Sepax GP-C18 column (4.6 × 250 mm, 5 µm) was used for the HPLC analysis. The injection volume was 10 µL, and the column temperature was maintained at 30 °C.

Small molecular components of toad venom. Fresh venom from toads from different areas was extracted with methanol and detected by HPLC according to the methods recommended by Cao et al. and the “Pharmacopoeia of the People’s Republic of China” [2].

Total proteins from the soluble fraction of toad venom. Samples were fractionated with a linear gradient of 5–90% acetonitrile acidified with 0.5% (*v*/*v*) trifluoroacetic acid for 105 min at a flow rate of 1.0 mL/min. The detection wavelength was set at 280 nm [13].

### 4.6. Cytotoxic Activity and Growth Rate Inhibition

The antiproliferative activity of the total protein of toad venom from HZ and LYG specimens against three selected cell lines was evaluated using an MTT assay, according to Mossman (1983). Briefly, HaCaT, MCF-7, and SGC-7901 cells (1 × 10^4^ cells/well) were seeded into 96-well plates and incubated with different concentrations of proteins extracted from toad venom (0.01, 0.1, 1, 10, and 100 µg/mL) for 48 h. The morphology of cells from each treatment was imaged using a Leica DM3000B microscope (Leica Microsystems GmbH, Frankfurt, Germany) [53].

Meanwhile, the cell growth rate in the presence and absence of the toad venom samples was compared, and the normalized growth rate inhibition (GR) was used to evaluate the effect of the samples on the cells [54]. Three cells (4 × 10^6^ cells/well) were seeded into 6-well plates and incubated with different concentrations of protein extracts (0.01, 0.1, 1, 10, 25, 50, 75, and 100 µg/mL) for 48 h. The cell count and death count were recorded when the extracts were present and absent, respectively. An online GR calculator (www.grcalculator.org) (accessed on 3 April 2023) was used for dose-response curves for GR value.

The HaCaT cell was provided by China Center for Type Culture Collection, CCTCC (Wuhan, China). The MCF-7 and SGC-7901 cells were obtained from the National Collection of Authenticated Cell Cultures (Shanghai, China).

### 4.7. Transcriptomic and Proteomic Analyses

Transcriptomic analysis and TMT-based proteomics analysis were conducted by Applied Protein Technology Co., Ltd (Shanghai, China). Briefly, total RNA was isolated using a TRIzol reagent kit (Takara Biomedical Technology Co., Ltd., Beijing, China) according to the manufacturer’s instructions. A cDNA library was constructed by reverse transcription and PCR amplification and sequenced on an Illumina HiSeq 2500 system. The raw reads were filtered using fastp software (version 0.20.0; HaploX Biotechnology Co., LTD, Guangzhou, China), and transcriptome assembly was performed using Trinity(http://trinityrnaseq.github.io) (accessed on 13 May 2022) [55,56]. The RNA sequencing data are available for download at the NCBI Sequence Read Archive (SRA) under the accession number PRJNA937175.

Proteins from each sample were trypsin-digested according to the filter-aided sample preparation method. The peptide mixture of each sample was labeled using the TMT reagent according to the manufacturer’s instructions (Thermo Scientific, Waltham, MA, USA). Then, fractionation was conducted via strong cation exchange resin chromatography using an AKTA purifier system (GE Healthcare, Chicago, IL, USA). Liquid chromatography-tandem mass spectrometry analysis was performed using a Q Exactive hybrid quadrupole orbitrap mass spectrometer (Thermo Scientific, Waltham, MA, USA). The raw data for each sample were searched using the MASCOT engine (version 2.2; Matrix Science, London, UK) embedded in Proteome Discoverer 1.4 software (Thermo Scientific, Waltham, MA, USA) for identification and quantitation analysis [57,58,59]. The proteomics data have been deposited to the ProteomeXchange Consortium via the iProX partner repository with accession number PXD040406.

### 4.8. Protein Identification and Bioinformatic Analysis

To analyze differences in protein expression among groups, a fold change of >1.2 or <0.83 and a *p*-value of <0.05 were used to identify up and downregulated proteins. NCBI Basic Local Alignment Search Tool (BLAST) + client software (NCBI-blast-2.2.28+-win32.exe, Bethesda, Maryland, USA) and InterProScan were used to find homolog sequences for the selected DEPs [58,60]. The GO, Pfam, KEGG, Non-Redundant, and Swiss-Prot databases were used to annotate all sequences using a BLAST search (http://blast.ncbi.nlm.nih.gov/Blast.cgi) (accessed on 1 November 2022) [56,61].

### 4.9. Antimicrobial Assay

*Escherichia coli*, *Bacillus subtilis*, and *Staphylococcus aureus* were plated on suitable solid medium plates. Recombinant Lysozyme C-1 solution (5 μL) with concentrations of 1, 0.1, and 0.01 μg/μL was added to the surface of the adhesive film on sterilized circular paper. PBS was used as the negative control, and 0.5 μg/μL ampicillin-kanamycin was used as the positive control [27,62]. Additionally, the protein supernatant of BL21(DE3) cells was used instead of the sample as a control.

### 4.10. Anti-Inflammatory and Analgesic Assays

ICR mice (18–22 g) and Sprague-Dawley rats (180–220 g) were purchased from the Laboratory Animal Center of Zhejiang Academy of Medical Sciences. All animal experiments were sanctioned by the Animal Ethics Committee of Zhejiang A&F University (ZAFUAC2022021), and all experimental procedures were performed in accordance with the Regulations for the Administration of Affairs Concerning Experimental Animals approved by the State Council of the People’s Republic of China.

Acetic acid-induced writhing assay. The acetic acid-induced writhing assay was performed according to an established protocol described by Nahida et al. and Deng et al. [49,63]. Sixty 4-week-old male ICR mice were randomly divided into six groups, including blank control (0.9% saline), model (0.9% saline), positive control (50 μg/kg aspirin), low-dosage NPB (50 μg/kg), medium-dosage NPB (100 μg/kg), and high-dosage NPB (200 μg/kg) groups. After 20 min, blank group mice were intraperitoneally injected with 0.9% saline, and the other groups were injected with 0.8% acetic acid. Then, the time of the first writhing and the writhing times of mice within 15 min after the first writhing were recorded. Each mouse was administered a dose of 10 mL/kg.

CFA-induced licking assay. According to Fattori et al. and Chen et al. [64,65], 48 six-week-old male SD rats were randomly divided into six groups, as in the acetic acid writhing experiment. Rats were treated once with 1, 2, or 10 ng of NPB, 0.5% aspirin, or 0.9% saline via an intrathecal route (between the L4 and L6 spinal segments, 10 μL) 10 min before intra-plantar injection of 0.9% saline or CFA (0.1 mL). The response time (paw licking or biting for the first time) and the paw licking times were determined within 0–15 min (first phase) and 15–30 min (second phase).

### 4.11. Statistical Analysis

Data are presented as the means ± standard deviation. Data analyses were performed with the SPSS 26.0 statistical package (IBM, Armonk, NY, USA). The Kolmogorov–Smirnov test was used to determine the normality and homogeneity of variance of all data [66,67,68]. For multi-group comparisons, *p* values were derived from one-way ANOVA or a Chi-square test. Statistical significance was set at *p* < 0.05.

## 5. Conclusions

Toad venom has been utilized to treat diseases in China for thousands of years. It contains a variety of effective compounds with different physiological activities and chemical components that have remarkable pharmacological effects. Recent pharmacological studies have demonstrated that the family Bufonidae produces numerous active peptides for a variety of biological functions such as antioxidant, antimicrobial, anticancer, angiogenesis-inhibiting, and wound healing activities. The existing quality evaluation standard of toad venom only focuses on small molecular components, while safety and quality evaluation standards for proteins and peptides are lacking. In this study, the content of small molecular components was negatively correlated with their protein components. Correlation analysis of transcriptomic and proteomic data showed the functional proteins and peptide distribution in the toad venom of *B. gargarizans*. Proteins were thoroughly analyzed, and a total of 18,897 unique peptides and 3724 quantified proteins were found. Meanwhile, a candidate list of functional proteins that could be potential quality markers was presented. Lysozyme C-1 and NPB were the most important functional proteins and could be used as high-quality markers. In the future, we are confident that the quality markers presented in this study can lay the foundation for quality control system studies of toad venom proteins, which will help to construct and improve the scientific and comprehensive quality evaluation method. Besides, the antimicrobial peptide Lysozyme C-1 and analgesic peptide NPB can be used as potential high-quality drugs, and the protein structure and activity mechanism can be studied.

## Figures and Tables

**Table 1:** Candidate list of potential quality markers: three types of functional proteins.

Accession	Annotation	Activity	Fold Change	Regulation
Comp52143_c1_seq21	Lysozyme C-1	Antimicrobial, Antitumor	3.1136	Up
Comp52065_c1_seq1	E3 ubiquitin-protein ligase MYCBP2	Antimicrobial, Antitumor	1.5784	Up
Comp25467_c0_seq2	Mindbomb E3 ubiquitin protein ligase 1	Antimicrobial	1.4762	Up
Comp52171_c0_seq2	Sushi, von Willebrand factor type A, EGF, and pentraxin domain-containing protein 1	Antimicrobial	1.4248	Up
Comp20546_c0_seq1	E3 ubiquitin-protein ligase HERC2	Antimicrobial, Antitumor	1.2580	Up
Comp59368_c0_seq1	RanBP-type and C3HC4-type zinc finger-containing protein 1-like, partial	Antimicrobial	1.2571	Up
Comp51468_c0_seq2	GLI pathogenesis-related 2	Antimicrobial	1.2232	Up
Comp47732_c0_seq1	GLI pathogenesis-related 2	Antimicrobial	1.2628	Down
Comp30628_c0_seq1	NHL repeat-containing protein 2	Antimicrobial	1.2907	Down
Comp50754_c1_seq2	Novel protein containing Sushi domain (SCR repeat) domain	Antimicrobial	1.3641	Down
Comp50084_c0_seq6	E3 ubiquitin-protein ligase listerin	Antimicrobial, Antitumor	1.6850	Down
Comp47859_c0_seq1	Cathepsin B	Antimicrobial	2.4604	Down
Comp43995_c0_seq5	E3 ubiquitin-protein ligase Makorin-2	Antimicrobial, Antitumor	8.7779	Down
Comp52833_c0_seq1	Secretory phospholipase A2 DsM-S1	Anti-inflammatory and Analgesic	2.0525	Down
Comp33502_c0_seq1	Neuropeptide B isoform 2 (NPB)	Anti-inflammatory and Analgesic	1.5847	Up
Comp52379_c0_seq11	Prostatic acid phosphatase	Anti-inflammatory and Analgesic	1.4639	Up
Comp80513_c0_seq1	Arylacetamide deacetylase-like 3	Anti-inflammatory and Analgesic	1.2288	Down
Comp40289_c0_seq1	Serpin B6	Antitumor	1.2113	Up
Comp39279_c0_seq1	Insulin-like growth factor-binding protein 2	Antitumor	1.3578	Down
Comp49982_c0_seq1	Neurotrypsin-like	Antitumor	1.2190	Up
Comp51288_c0_seq3	Leucine-rich repeat neuronal protein 1	Antitumor	1.2108	Up
Comp44678_c0_seq2	Galectin-9	Antitumor	1.6774	Down
Comp51969_c1_seq1	Transportin 3	Antitumor	1.2704	Down
Comp47586_c0_seq2	Immunoglobulin light chain type III	Antitumor	1.3508	Down
Comp22487_c0_seq1	Glutathione S-transferase Mu 6	Antitumor	1.3454	Up
Comp70215_c0_seq1	Cytochrome P450 2C41	Antitumor	1.9139	Up
Comp23296_c0_seq1	Proteasome assembly chaperone 4	Antitumor	1.2332	Down
Comp48235_c2_seq1	Protein tyrosine phosphatase type IVA 3	Antitumor	1.2844	Up
Comp702_c0_seq1	Cytochrome P450 20A1	Antitumor	1.2398	Up
Comp44914_c0_seq1	Proteasome assembly chaperone 3	Antitumor	1.2478	Up
Comp35535_c0_seq1	DDB1- and CUL4-associated factor 11 homolog	Antitumor	1.2019	Up
Comp68566_c0_seq1	Cytochrome P450, family 20, subfamily A, polypeptide 1	Antitumor	1.2497	Down

## Data Availability

The original contributions presented in the study are publicly available in the submitted databases. The raw data are available upon a reasonable request from the corresponding author.

## Supplementary Materials

The following supporting information can be downloaded at: https://www.mdpi.com/article/10.3390/molecules28083628/s1, Table S1: Information about the potential quality markers.

## Sample Availability

Samples of the compounds are not available from the authors.
