# Supplementary material for: Identification of Protein Quality Markers in Toad Venom from Bufo gargarizans
Source: Molecules. 2023 Apr 21;28(8):3628. doi: 10.3390/molecules28083628 (PMC10141085; doi:10.3390/molecules28083628)
Supplement: Supplementary file 1 [file molecules-28-03628-s001.zip › molecules-2312105-supplementary.pdf]

Table S1. More information on the potential quality markers candidate list.

Accession	Swiss-ID	Protein	Function
Comp52143_c1_seq21	P12067	Lysozyme C-1	Antifungal effect of Candida krusei and Candida albicans
Comp52065_c1_seq1	O75592	E3 ubiquitin-protein ligase MYCBP2	Correlated with inflammatory cell infiltration and cancer prognosis of thyroid cancer
Comp25467_c0_seq2	S4RQT6	Mindbomb E3 ubiquitin protein ligase 1	Antiviral action; anti-angiogenic factor
Comp52171_c0_seq2	A2AVA0	Sushi, von Willebrand factor type A, EGF and pentraxin domain-containing protein 1	Play a role in the cell attachment process
Comp20546_c0_seq1	O95714	E3 ubiquitin-protein ligase HERC2	Regulates ubiquitin-dependent retention of repair proteins on damaged chromosomes
Comp59368_c0_seq1	A9JTG5	RanBP-type and C3HC4-type zinc finger-containing protein 1	Interferes with TNF-induced cell death and thereby prevents inflammation
Comp51468_c0_seq2<br>Comp47732_c0_seq1	Q5U4W9	GLI pathogenesis-related 2 S homeolog	Suppressed growth of lung cancer cells and lung tumor xenografts
Comp30628_c0_seq1	A4IF69	NHL repeat-containing protein 2	Enhance myofibroblast differentiation in FINCA disease
Comp50754_c1_seq2	O02839	Membrane cofactor protein	Protects autologous cells against complement-mediated injury
Comp50084_c0_seq6	A0JM49	E3 ubiquitin-protein ligase listerin	Extraction and degradation of the incomplete translation product
Comp47859_c0_seq1	A1E295	Cathepsin B	Implicated in tumor invasion and metastasis
Comp43995_c0_seq5	B0F0H3	E3 ubiquitin-protein ligase Makorin-2	Catalyze the covalent attachment of ubiquitin moieties onto substrate proteins; inhibits neurogenesis and axis formation
Comp52833_c0_seq1	A8CG84	Basic phospholipase A2 DsM-S1	Catalyzes the calcium-dependent hydrolysis of the 2-acyl groups in 3-sn-phosphoglycerides; effect on nerves
Comp33502_c0_seq1	Q8K4P1	Neuropeptide B	Regulation of pain
Comp52379_c0_seq11	A6H730	Prostatic acid phosphatase	Lipid phosphatase activity and inactivates lysophosphatidic acid in seminal plasma
Comp80513_c0_seq1	A2A7Z8	Arylacetamide deacetylase-like 3	Showing features for active site
Comp40289_c0_seq1	O02739	Serpin B6	Inhibition of serine proteinases

Accession	Swiss-ID	Protein	Function
			present in the brain or extravasated from the blood
Comp39279_c0_seq1	A4IIA2	Insulin-like growth factor-binding protein 2	Inhibit or stimulate the growth promoting effects of the IGFs on cell culture
Comp49982_c0_seq1	O08762	Neurotrypsin	Plays a role in neuronal plasticity and the proteolytic action
Comp51288_c0_seq3	A0N0X6	Leucine-rich repeat neuronal protein 1	Suppresses apoptosis of gastric cancer cells through regulation of Fas/FasL
Comp44678_c0_seq2	O00182	Galectin-9	Promotes ability of mesenchymal stromal cells to suppress T-cell proliferation; expands regulatory T-cells and induces cytotoxic T-cell apoptosis following virus infection. Restricts intracellular bacterial growth
Comp51969_c1_seq1	Q9Y5L0	Transportin 3	Involved in immunodeficiency virus (HIV-1) infection
Comp47586_c0_seq2	A0M8Q6	Immunoglobulin lambda constant 7	Results in the elimination of bound antigens
Comp22487_c0_seq1	O35660	Glutathione S-transferase Mu 6	Detoxifies reactive metabolites of xenobiotics
Comp70215_c0_seq1	O62671	Cytochrome P450 2C41	Catalyze the metabolism of a wide range of chemicals
Comp23296_c0_seq1	P0C7N9	Proteasome assembly chaperone 4	responsible for degradation of ubiquinated proteins in eukaryotic cells
Comp48235_c2_seq1	A2VDT1	Protein tyrosine phosphatase type IVA 3	Associated with cell proliferation, cell motility and invasive activity
Comp702_c0_seq1	Q5E980	Cytochrome P450 20A1	Play a neurophysiological role
Comp44914_c0_seq1	A7SP74	Proteasome assembly chaperone 3	Enables its effective degradation via chaperone-mediated autophagy
Comp35535_c0_seq1	A8XEN7	DDB1- and CUL4-associated factor 11 homolog	Involved in regulation of lifespan
Comp68566_c0_seq1	Q6YTF5	Cytochrome P450 76M5	Play a pivotal role in the detoxification of xenobiotics, cellular metabolism and homeostasis
